# Nuclear ErbB2 represses DEPTOR transcription to inhibit autophagy in breast cancer cells

**DOI:** 10.1038/s41419-021-03686-9

**Published:** 2021-04-14

**Authors:** Yanli Bi, Longyuan Gong, Pengyuan Liu, Xiufang Xiong, Yongchao Zhao

**Affiliations:** 1 Department of Hepatobiliary and Pancreatic Surgery, the First Affiliated Hospital, Zhejiang University School of Medicine, Hangzhou, China; 2 Zhejiang Provincial Key Laboratory of Pancreatic Disease, the First Affiliated Hospital, Zhejiang University School of Medicine, Hangzhou, China; 3 Institute of Translational Medicine, Zhejiang University School of Medicine, Hangzhou, China; 4 Cancer Institute of the Second Affiliated Hospital, Zhejiang University School of Medicine, Hangzhou, China; 5 Cancer Center, Zhejiang University, Hangzhou, China

**Keywords:** Macroautophagy, Transcriptional regulatory elements

## Abstract

ErbB2, a classical receptor tyrosine kinase, is frequently overexpressed in breast cancer cells. Although the role of ErbB2 in the transmission of extracellular signals to intracellular matrix has been widely studied, the functions of nuclear ErbB2 remain largely elusive. Here, we report a novel function of nuclear ErbB2 in repressing the transcription of DEPTOR, a direct inhibitor of mTOR. Nuclear ErbB2 directly binds to the consensus binding sequence in the *DEPTOR* promoter to repress its transcription. The kinase activity of ErbB2 is required for its nuclear translocation and transcriptional repression of *DEPTOR*. Moreover, the repressed DEPTOR by nuclear ErbB2 inhibits the induction of autophagy by activating mTORC1. Thus, our study reveals a novel mechanism for autophagy regulation by functional ErbB2, which translocates to the nucleus and acts as a transcriptional regulator to suppress *DEPTOR* transcription, leading to activation of the PI3K/AKT/mTOR pathway to inhibit autophagy.

## Introduction

The mammalian target of rapamycin (mTOR), an evolutionarily conserved serine/threonine protein kinase, serves as a central regulator of cell growth, proliferation, survival, and autophagy, and is frequently activated in many human cancers^1^. DEPTOR (DEP-domain containing mTOR-interacting protein), a naturally occurring inhibitor of mTOR, directly binds to mTOR and suppresses the kinase activity of mTOR complex 1 (mTORC1) and 2 (mTORC2). Thus, DEPTOR knockdown increases the activity of both mTORC1 and mTORC2, thereby promoting cell proliferation and survival. Under certain circumstances, DEPTOR overexpression inhibits S6K1, an mTORC1 substrate, and alleviates S6K-mediated IRS-1/PI3K (phosphoinositide 3 kinase)-dependent negative feedback to activate AKT, leading to cell survival, which facilitates tumor progression^2–4^. Thus, DEPTOR levels need be precisely regulated.

ErbB2, also known as Her2 or Neu, is a member of the epidermal growth factor receptor family, which is composed of an extracellular domain, a single transmembrane helix, and a kinase domain^5^. Till date, no ligands for ErbB2 have been identified. Upon stimulation, ErbB2 can only be recruited as a co-receptor to form heterodimers with other members of the ERBB family, such as ErbB1 and ErbB3, and may also form homodimers when it is overexpressed, leading to the phosphorylation and activation of the ErbB2 kinase domain^5,6^. Activated ErbB2 then conveys the extracellular signals to activate intracellular downstream signaling pathways, including the PI3K/AKT/mTOR pathway and the mitogen-activated protein kinase (MAPK) pathway, leading to cell proliferation, survival, and invasion^5^. *ERBB2* gene is overexpressed in 20–30% of breast cancer cases, which correlates with poor prognosis, lymph-node metastasis, and relative resistance to some drugs^6^. Thus, ErbB2 has served as a biomarker for breast cancer prognosis and a therapeutic target for cancer treatment^5^. Recently, accumulating evidences showed that ErbB2 shuttles into the nucleus and plays important roles in a variety of cellular processes, such as proliferation, signal transduction, and resistance to cancer therapy^7–16^. Mechanistically, ErbB2 contains a putative nuclear localization signal (NLS), which is located adjacent to the transmembrane domain. ErbB2 forms a complex with importin-β through the ErbB2-NLS, in which importin-β binds to nucleoporins of nuclear pore complexes and leads to ErbB2 nuclear translocation^10,11,17^.

In this study, we report that nuclear ErbB2, acting as a transcription repressor, directly binds to the consensus binding site of the *DEPTOR* promoter and transcriptionally represses *DEPTOR* expression, resulting in mTORC1/2 activation. Moreover, ErbB2 activation via autophosphorylation promotes its nuclear translocation and subsequent inhibition of DEPTOR expression to regulate autophagy. Thus, our study uncovered a novel mechanism for ErbB2 in regulation of mTOR signaling and autophagy induction by translocating to the nucleus and directly repressing DEPTOR transcription.

## Results

### ErbB2 negatively regulates DEPTOR transcription

**Fig. 1:**
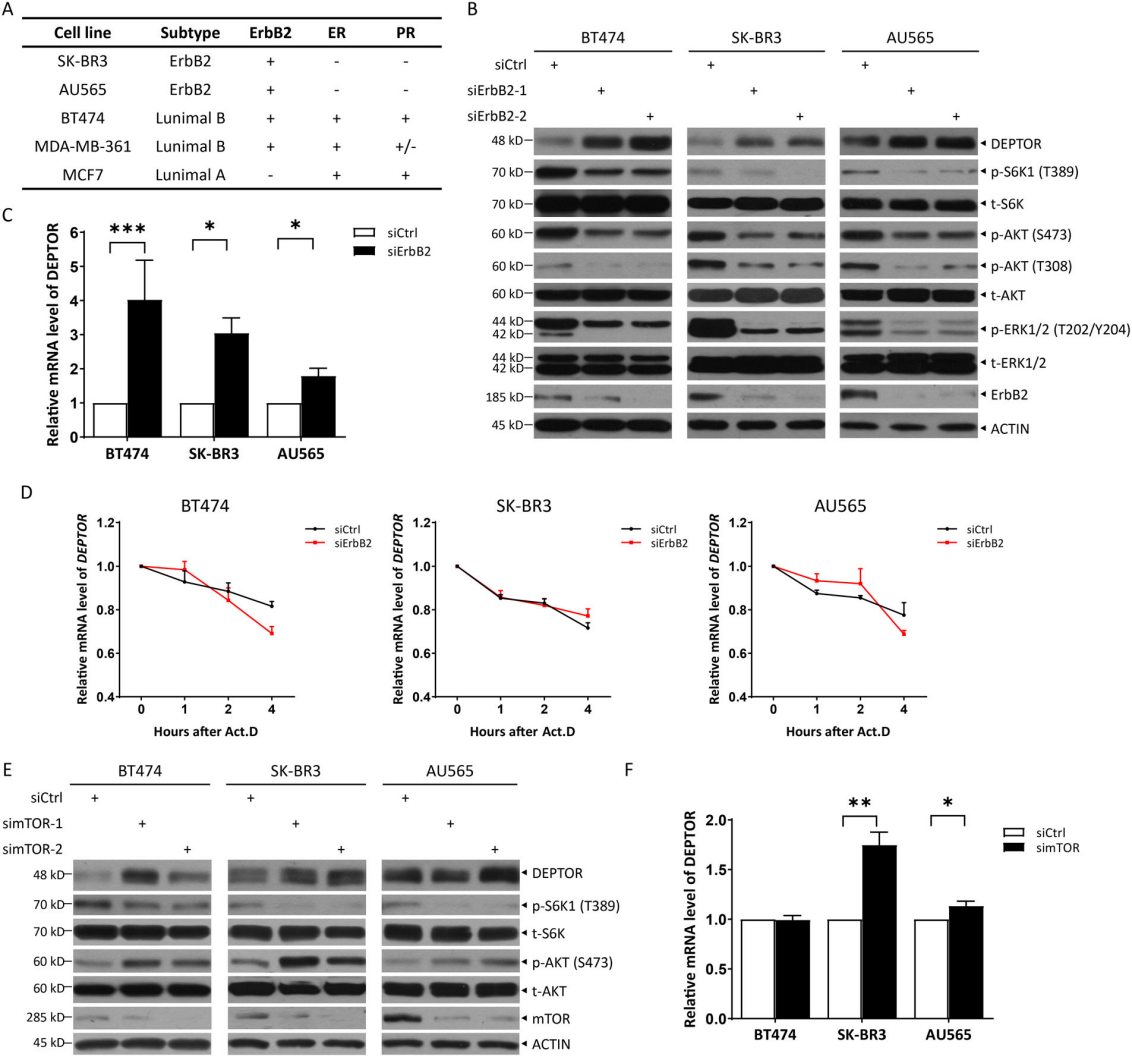
DEPTOR is negatively regulated by ErbB2 at transcription levels. **A** The molecular characteristics of breast cancer cells. **B–D** ErbB2 knockdown significantly induced the expression of DEPTOR at transcriptional levels: BT474, SK-BR3, and AU565 cells were transfected with siRNA targeting ErbB2 or scrambled control siRNA, and then subjected to western blotting (**B**) or qRT-PCR analysis (**C**, *n* = 3); or treated with 5 μg/ml of actinomycin D (Act. D) for indicated time periods, and then subjected to qRT-PCR analysis (**D**, *n* = 3). **E**, **F** mTOR silencing moderately increased the expression of DEPTOR: BT474, SK-BR3, and AU565 cells were transfected with siRNA targeting mTOR or scrambled control siRNA, and then subjected to western blotting (**E**) or qRT-PCR analysis (**F**, *n* = 3). Data from three independent experiments were expressed as mean ± SEM, *p < 0.05, **p < 0.01, ***p < 0.001.

ErbB2 is an oncoprotein that promotes cell growth and survival by activating the PI3K/AKT/mTOR and RAS/ERK pathways^5^. To further determine the mechanism underlying the regulation of these pathways by ErbB2, we transfected two different ErbB2 siRNA oligos into three well-known ErbB2 positive breast cancer cell lines, BT474, SK-BR3, and AU565 (Fig. 1A). Surprisingly, we found a significant accumulation of DEPTOR, a naturally occurring inhibitor of mTOR^2^ (Fig. 1B). Consistently, ErbB2 silencing reduced the phosphorylation of S6K1 and AKT, the downstream effectors of mTORC1 and mTORC2, respectively, suggesting that ErbB2 knockdown may inhibit the activation of mTORC1 and mTORC2 by DEPTOR induction (Fig. 1B). Further, we found that ErbB2 depletion dramatically increased the mRNA levels of DEPTOR in all the tested cells (Fig. 1C). To exclude the possibility that the increase in mRNA levels of DEPTOR upon ErbB2 knockdown was due to stabilization of DEPTOR mRNA, we simultaneously treated cells with actinomycin D, a RNA synthesis inhibitor^18^, and found that ErbB2 knockdown had no effect on the mRNA half-lives of DEPTOR (Fig. 1D). Thus, these results suggest that ErbB2 inactivation promotes the transcription of DEPTOR mRNA, but has no effect on its mRNA stability.

Given that mTORC1 and mTORC2 have been shown to negatively regulate DEPTOR at the mRNA as well as protein levels^2^, we compared the effect of ErbB2 depletion on DEPTOR levels with that of mTOR depletion by silencing mTOR via two different siRNA oligos. We found that mTOR depletion had a moderate effect on DEPTOR protein levels (Fig. 1E). Consistently, it was observed that mTOR silencing induced DEPTOR transcription (Fig. 1F) by less than two folds in SK-BR3 cells; whereas only slight induction and no induction was observed in AU565 cells and BT474 cells, respectively. Thus, DEPTOR induction by ErbB2 silencing (Fig. 1B, C) was much higher than that by mTOR silencing (Fig. 1E, F), indicating that ErbB2 may directly suppress DEPTOR expression.

### ErbB2 translocates to the nucleus and directly binds to *DEPTOR* promoter to repress its transcription

**Fig. 2:**
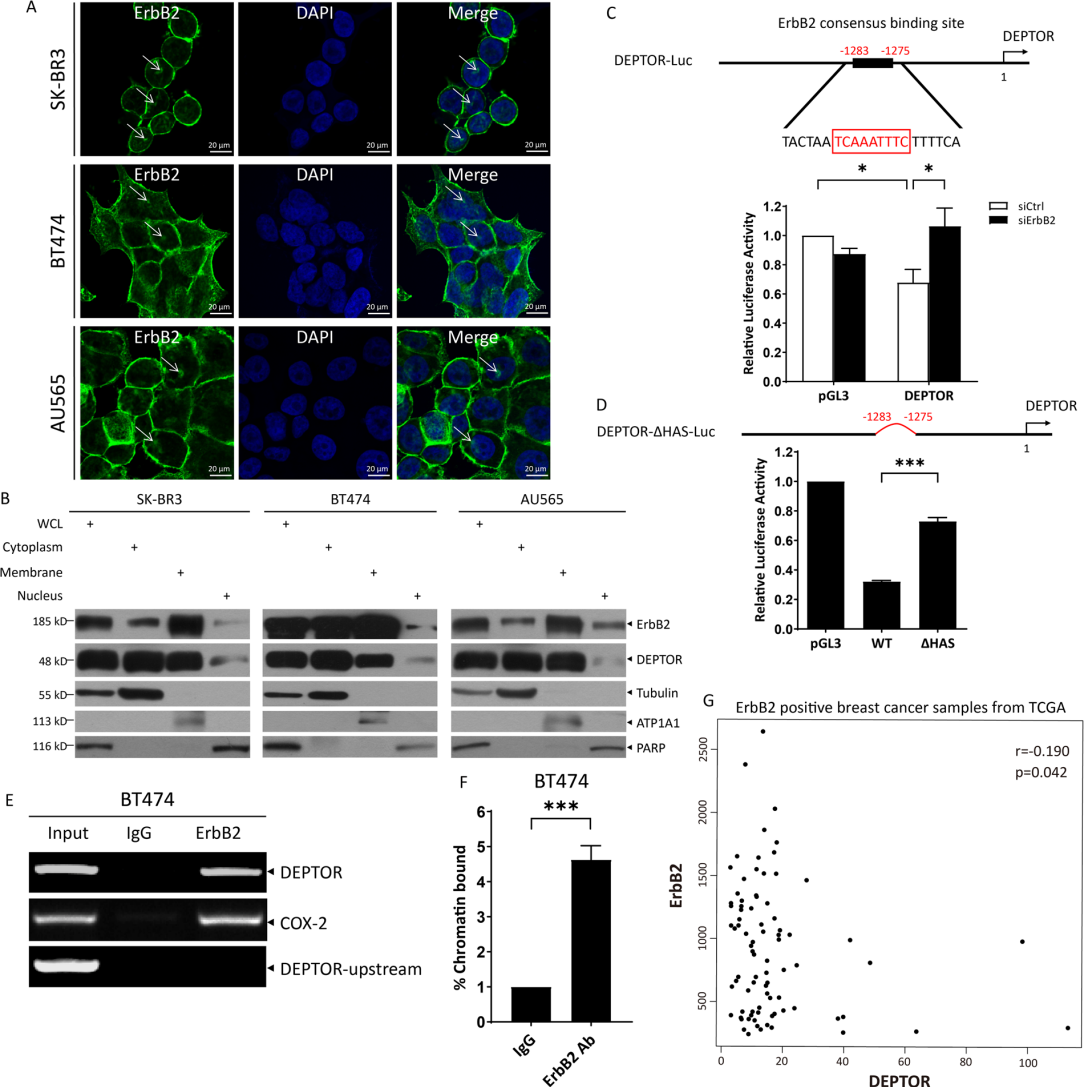
ErbB2 is translocated to the nucleus and binds to HAS in the *DEPTOR* promoter. **A**, **B** Nuclear translocation of ErbB2: SK-BR3, BT474, and AU565 cells were stained with anti-ErbB2 antibody and photographed using Nikon confocal microscopy (**A**), or harvested for subcellular fractionation, followed by western blotting (**B**). Scale bars represent 20 μm. Tubulin, ATP1A1, and PARP were used as cytoplasmic, plasmalemmal, and nuclear markers, respectively. **C**, **D** Silencing of ErbB2 or deletion of HAS increases DEPTOR transcription. Shown are luciferase reporters under the control of DEPTOR promoter containing the ErbB2 consensus binding site (HAS, −1283 to −1275) (**C**) and with HAS deleted (deleting −1283 to −1275) (**D**). SK-BR3 cells were cotransfected with the indicated siRNA oligos and luciferase reporter construct (DEPTOR-Luc) for 48 h (**C**), BT474 cells were transfected with the indicated luciferase reporter constructs for 24 h (**D**), followed by luciferase reporter assay. Data from three independent experiments, each run in duplicates, were expressed as mean ± SEM, *p < 0.05, ***p < 0.001. **E**, **F** ErbB2 binds to the *DEPTOR* promoter: fresh BT474 cells were harvested for the ChIP assay with anti-ErbB2 antibody or normal IgG, followed by PCR amplification of indicated promoter fragments (**E**), or quantitative real-time PCR analysis (**F**). The HAS of COX-2 and the fragment upstream of DEPTOR “start codon” (−6586 to −6449, DEPTOR-upstream) were used as positive and negative controls, respectively. **G** Pearson’s correlation between the expression of ErbB2 and DEPTOR in ErbB2-positive BRCA tumor tissues.

It has been previously shown that ErbB2 binds to the consensus Her2-binding site (HAS, HER-2-associated sequence) in the *COX-2* promoter and transactivates its transcription in the nucleus^7^. Therefore, we hypothesized that ErbB2 suppresses DEPTOR expression by directly regulating its transcription. We first determined that ErbB2 was indeed located in the nucleus of SK-BR3, BT474, and AU565 cells by immunofluorescence staining using an anti-ErbB2 antibody^7,8,14^ (Fig. 2A). Moreover, ErbB2 was readily detected in the nuclear fractions of SK-BR3, BT474, and AU565 cells (Fig. 2B). Next, we performed bioinformatics analysis of the *DEPTOR* promoter and identified a putative HAS (TCAAATTTC) at −1283 to −1275, located upstream from the “start” codon of *DEPTOR* (Fig. 2C). To determine if the HAS plays a role in ErbB2-regulated *DEPTOR* transcription, we first constructed a luciferase reporter under the control of *DEPTOR* promoter containing HAS (DEPTOR-Luc). We then performed luciferase reporter assay and found that ErbB2 silencing relieved the inhibition of *DEPTOR* transcription (Fig. 2C). Next, we constructed another luciferase reporter with the deletion of this putative HAS (DEPTOR-ΔHAS-Luc) (Fig. 2D), and found that the inhibition of luciferase activity by DEPTOR-Luc was partially abolished by deletion of HAS (Fig. 2D). More importantly, ErbB2 directly bound to the fragment of *DEPTOR* promoter containing HAS, as demonstrated by chromatin immunoprecipitation (ChIP) assay (Fig. 2E, F). The HAS of COX-2 and another fragment upstream of DEPTOR “start codon” (−6586 to −6449, DEPTOR-upstream) were used as positive and negative controls, respectively (Fig. 2E). In addition, we analyzed RNA-seq expression of ErbB2 and DEPTOR in ErbB2-positive BRCA tumor tissues from TCGA. A moderate negative correlation between ErbB2 and DEPTOR (*r* = −0.190, *p* = 0.042) was found (Fig. 2G). Altogether, our results suggest that nuclear ErbB2 directly binds to the consensus HAS in the *DEPTOR* promoter and transcriptionally represses DEPTOR expression.

### ErbB2 kinase activity is required for its nuclear translocation and transcriptional repression of DEPTOR

**Fig. 3:**
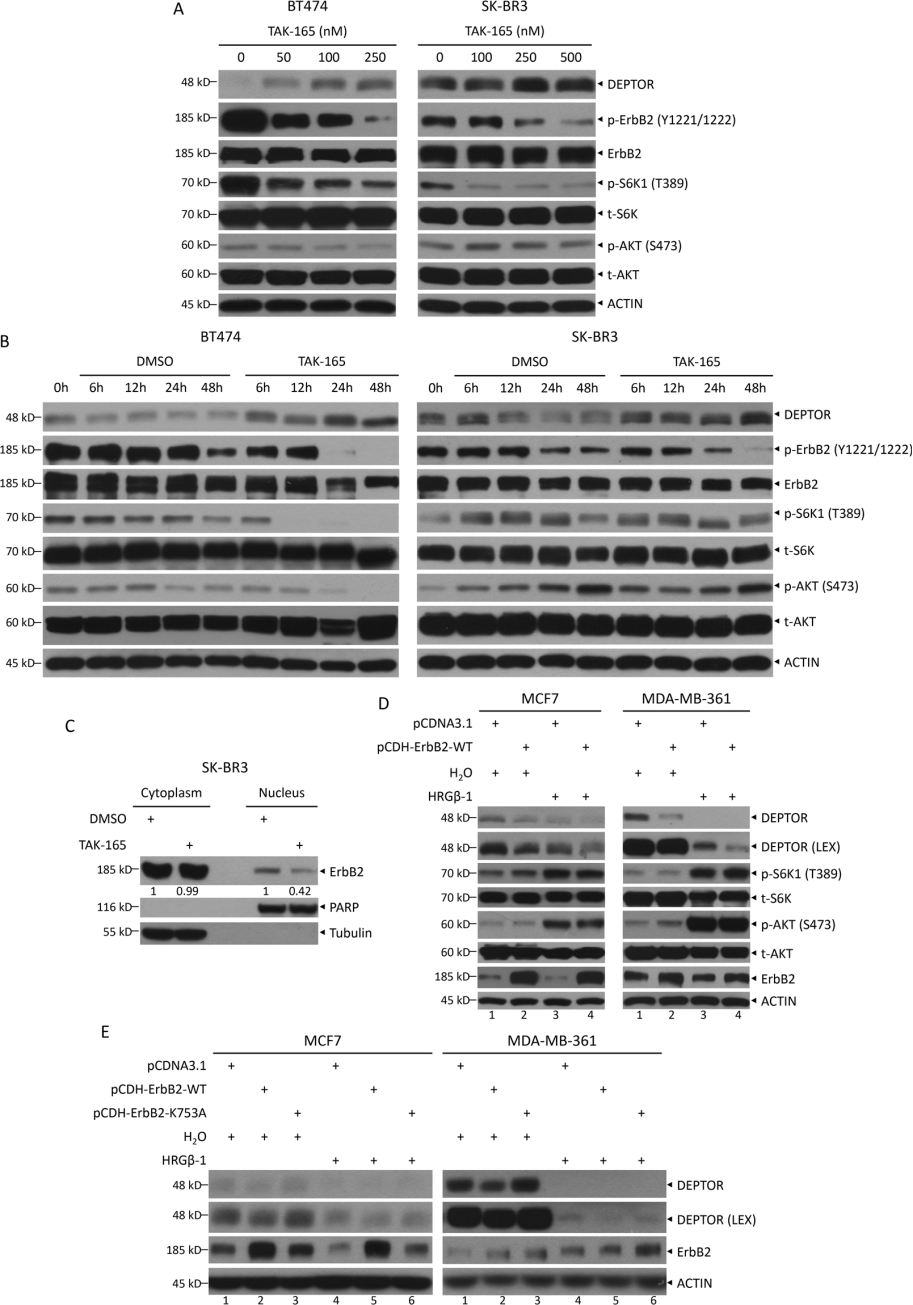
The kinase activity of ErbB2 is required for ErbB2 nuclear translocation and transcriptional repression of DEPTOR. **A** Inhibition of ErbB2 by TAK-165 induces DEPTOR levels in a dose-dependent manner: BT474 and SK-BR3 cells were treated with various doses of the specific ErbB2 kinase inhibitor TAK-165 for 24 h, followed by western blotting with the indicated antibodies. **B** TAK-165 treatment induces DEPTOR levels in a time-dependent manner: BT474 and SK-BR3 cells were treated with 0.1 μM or 0.25 μM TAK-165 for various time periods, followed by western blotting with the indicated antibodies. **C** TAK-165 treatment reduces ErbB2 nuclear translocation: SK-BR3 cells were treated with TAK-165 for 48 h and then subjected to nuclear fractionation, followed by western blotting with the indicated antibodies. The band density was quantified by ImageJ software and expressed as relative gray value, by setting the control value to 1. **D**, **E**. Wild-type, not kinase dead mutant, ErbB2 represses DEPTOR expression. Cells were transfected with indicated plasmids, and treated with or without 40 ng/ml of HRGβ-1 for 12 h, and then, subjected to IB with indicated Abs. LEX longer exposure.

Previous studies showed that inhibiting the kinase activity of ErbB2 or disrupting its nuclear localization suppresses the binding to the promoter of its targeting genes^7,19^. To determine whether the tyrosine kinase activity of ErbB2 is required for its nuclear translocation and DEPTOR repression, we treated cells with TAK-165, a specific ErbB2 kinase inhibitor^20^ and found that TAK-165 caused a dose- (Fig. 3A) and time-dependent (Fig. 3B) inhibition of ErbB2 autophosphorylation, indicating the inhibition of ErbB2 kinase activity. Importantly, DEPTOR was also induced in a dose- (Fig. 3A) and time-dependent (Fig. 3B) manner upon TAK-165 treatment, suggesting the requirement of ErbB2 kinase activity for DEPTOR repression. We further investigated whether ErbB2 activation promotes its nuclear translocation, and found that TAK-165 treatment suppressed nuclear translocation of ErbB2 (Fig. 3C). Conversely, we overexpressed ErbB2 in ErbB2 relatively low-expressing MCF7 and MDA-MB-361 cells, and found a consequent reduction of DEPTOR (Fig. 3D, lanes 2 vs 1). Moreover, the treatment of HRGβ-1, a ligand binding ErbB3 to stimulate the formation of ErbB2/ErbB3 dimers and activate ErbB2^13^, not only decreased DEPTOR significantly in cells transfected with mock vector (Fig. 3D, lanes 3 vs 1), but also further reduced DEPTOR levels in ErbB2-overexpressed cells (Fig. 3D, LEX, lanes 4 vs 3). Finally, we overexpressed a kinase dead mutant ErbB2 (K753A), and found this mutation impaired the ability to reduce DEPTOR expression regardless of HRGβ-1 treatment (Fig. 3E, lanes 3 vs 2 and 1, lanes 6 vs 5 and 4). Altogether, the results suggest that activation of ErbB2 via phosphorylation is critical for the nuclear translocation of ErbB2 and consequent repression of DEPTOR transcription.

### ErbB2 inactivation induces autophagy through the induction of DEPTOR

**Fig. 4:**
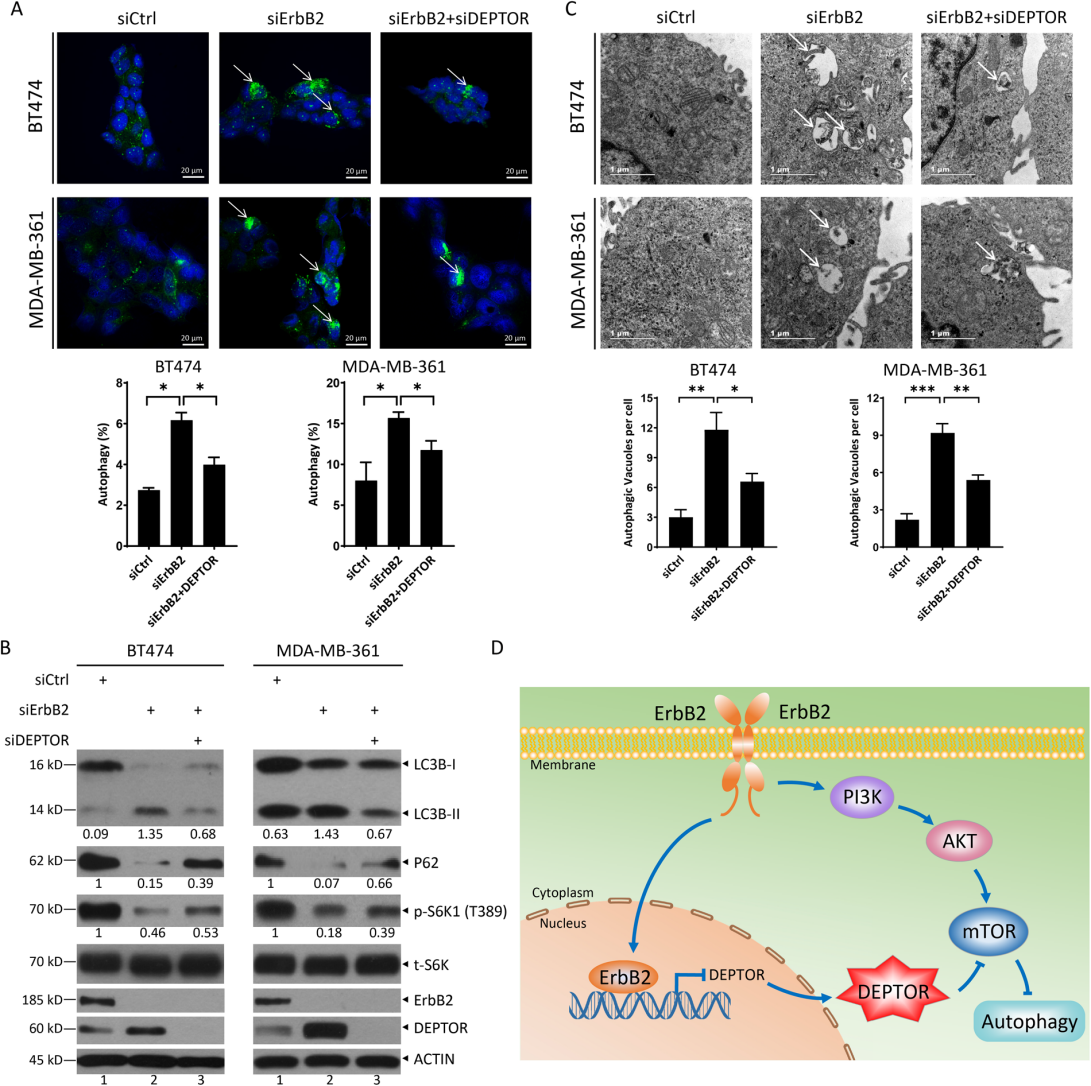
ErbB2 knockdown induces autophagy through the induction of DEPTOR. **A** Autophagy was measured by appearance of punctate vesicle structure: BT474 and MDA-MB-361 cells were transfected with indicated siRNA for 48 h, and then subjected to immunofluorescence and photography under Nikon confocal microscopy. Scale bars represent 20 μm. Cells with punctate vesicle structures of LC3 were counted and expressed as percentage of autophagy (bottom). *p < 0.05. **B** Autophagy was measured by LC3 conversion and p62 degradation: BT474 and MDA-MB-361 cells were transfected with indicated siRNA, followed by IB with indicated antibodies. The band density was quantified using Image J and expressed as relative gray value, compared with the control, by arbitrarily setting the control value as 1. **C** Autophagosomes detected by transmission electron microscopy (TEM). BT474 and MDA-MB-361 cells were transfected with indicated siRNA for 48 h, followed by TEM analysis. Autophagosomes were indicated by arrows. Direct magnification: ×30 000. Scale bars represent 1 μm. Autophagosomes were counted and expressed as autophagic vacuoles per cell (bottom). *p < 0.05, **p < 0.01, ***p < 0.001. **D** A model for repression of DEPTOR transcription by nuclear ErbB2 to activate mTOR pathway and suppress autophagy. See text for details.

Several studies clearly demonstrated that DEPTOR induces significant autophagy by mTORC1 inactivation^21–28^. Furthermore, ErbB2 blocks autophagy initiation by directly interacting with Beclin 1, the mammalian orthologue of yeast Atg6 that plays a central role in autophagy^29,30^. Thus, we next determined whether DEPTOR induced by ErbB2 inactivation contributes to autophagy induction upon ErbB2 depletion. Indeed, ErbB2 knockdown effectively induced autophagy, as reflected by the autophagic punctate structures in immunofluorescence staining of endogenous LC3B (Fig. 4A, middle panels), the conversion of LC3-I to LC3-II and p62 degradation (Fig. 4B, lanes 2 vs 1), and detectable autophagosomes by electron microscopy (Fig. 4C, middle panels). Meanwhile, DEPTOR was induced significantly along with the inactivation of mTORC1 upon ErbB2 silencing, as reflected by decreased S6K1 phosphorylation (Fig. 4B, lanes 2 vs 1). Interestingly, simultaneous knockdown of DEPTOR partially restored mTORC1 activity (Fig. 4B, lanes 3 vs 2 and 1). More importantly, simultaneous silencing of DEPTOR remarkably reduced autophagic punctate structures (Fig. 4A, right panels), partially inhibited the conversion of LC3-I to LC3-II and p62 degradation (Fig. 4B, lanes 3 vs 2 and 1), and decreased the numbers of autophagosomes (Fig. 4C, right panels), compared to ErbB2 silencing only, indicating a causal role of DEPTOR in autophagy induction by ErbB2 knockdown. Taken together, the results clearly demonstrated that ErbB2 inactivation induces autophagy by inducing DEPTOR to inactivate mTORC1.

## Discussion

In this study, we proved that nuclear ErbB2 represses DEPTOR transcription to effectively block autophagy induction by the following lines of evidence: (1) ErbB2 silencing induced DEPTOR levels at the mRNA as well as protein levels; (2) ErbB2 was readily detected in the nucleus of multiple breast cancer cells, and directly bound to the HAS in the *DEPTOR* promoter; (3) silencing of ErbB2 or deletion of HAS in the *DEPTOR* promoter relieved the inhibition of luciferase activity of the *DEPTOR* promoter; (4) inhibition of ErbB2 kinase activity reduced nuclear translocation of ErbB2, resulting in DEPTOR induction; and (5) ErbB2 silencing induced obvious autophagy, which was partially rescued by simultaneous knockdown of DEPTOR.

It has been previously shown that mTOR negatively regulates DEPTOR at the transcriptional and post-translational levels^2,21,22,31^. In addition, DEPTOR transcription is positively regulated by Notch1 in T cell leukemogenesis^32^, glucocorticoids in a glucocorticoid receptor-dependent manner during adipogenesis^33^, and Baf60c-Six4 in skeletal muscles^34^. Our study identified the first transcriptional suppressor, ErbB2, which negatively regulates DEPTOR expression. Interestingly, the induction of DEPTOR at protein and mRNA levels by ErbB2 knockdown was much greater than that by mTOR silencing (Fig. 1). Thus, ErbB2 appears to play a significant role in suppressing DEPTOR transcription in ErbB2 positive breast cancer cells.

Previous studies have shown that ErbB2 is located in the nucleus and acts as a transcription regulator to activate the transcription of *COX-2* by directly binding to the HAS in its promoter^7^, or to enhance rRNA synthesis by promoting the binding of RNA Pol I to rDNA^8^, etc. Further studies revealed that nuclear ErbB2 is involved in breast cancer cell growth, metastasis, and drug resistance, and it is being validated as a novel therapeutic target in ErbB2-positive breast cancer^11^. In this study, we report that nuclear ErbB2 serves as a transcriptional suppressor, as evidenced by the enhancement in transcriptional activity of the *DEPTOR* promoter upon ErbB2 silencing (Fig. 2C) or deletion of HAS (Fig. 2D). This transcription suppressing activity might be determined by the interaction of ErbB2 with certain proteins in the nucleus, which needs to be further explored. Likewise, the transcriptional regulation of both *DEPTOR* and *COX-2* requires the kinase activity of ErbB2. It has been previously shown that functional ErbB2 is required for binding to the *COX-2* promoter^7^. In our study, inhibition of ErbB2 by its specific inhibitor TAK-165 reduced ErbB2 levels in the nucleus (Fig. 3C), but increased DEPTOR levels (Fig. 3A, B). These results implied that the increase in DEPTOR levels upon TAK-165 treatment might be attributed to the decrease in ErbB2 nuclear translocation (Fig. 3C). Furthermore, ErbB2 inactivation induced DEPTOR to inhibit mTORC1, subsequently leading to autophagy induction (Fig. 4). Thus, the inhibition of mTOR by DEPTOR may contribute to the therapeutic role of targeting nuclear ErbB2.

In addition, previous studies have shown that ErbB2 can block autophagy initiation by modulation of Beclin 1 in breast cancer and Alzheimer’s disease^29,30^, and regulate autophagic cell death by modulation of ATG4B expression in retinal pigment epithelium cells^35^. The incidence of mammary tumors triggered by mammary-specific overexpressing activated ErbB2 was obviously lower in mice carrying a knock-in mutant Becn1^F121A/F121A^ with increased autophagy, compared to Becn1^WT/WT^ mice, and these mutant Becn1^F121A/F121A^ mice had significant longer life span, demonstrating that ErbB2-mediated autophagy suppression facilitates breast tumorigenesis^30^. Interestingly, ErbB2-mediated autophagy suppression also renders ErbB2-induced breast tumorigenesis in a Beclin 1-independent manner^36^. It is worth to investigate whether ErbB2-induced breast tumorigenesis is mediated by autophagy suppression via DEPTOR repression. Moreover, an autophagy-inducing peptide totally suppressed the growth of ErbB2-positive xenografts^30^, implying that targeting ErbB2-mediated autophagy suppression might represent an attractive approach for ErbB2-positive breast cancer therapy. Consistently, the induced autophagy contributes to the efficacy of ErbB2-targeted therapies^37^, enhancing tumor cells killing^38^, paradoxically, or facilitating drug resistance^39,40^. The complex roles of autophagy in ErbB2-targeted therapies warrant further investigation. In our study, we found that ErbB2 inactivation dramatically induced autophagy by inducing DEPTOR in ErbB2-positive breast cancer cells (Figs. 1B, 3A, B, 4A, B). Thus, whether and how autophagy regulated by the ErbB2-DEPTOR axis contributes to ErbB2-targeted therapies is an interesting topic to further explore using mouse model of breast cancer. Finally, given that DEPTOR is a direct inhibitor of mTORC1 and mTORC2^4^, induced DEPTOR upon ErbB2 inactivation not only promoted autophagy, but also might mediate cell proliferation, survival, and drug resistance^21,22^. Thus, targeting DEPTOR may be an attractive approach to overcome the resistance of ErbB2-targeted therapies.

In summary, our study identified the mTOR inhibitor, DEPTOR, as a novel downstream target of nuclear ErbB2. In addition to activation of the PI3K/AKT/mTOR pathway in a classical way in response to extracellular signals, ErbB2 is translocated to the nucleus, where it represses DEPTOR transcription to further activate the PI3K/AKT/mTOR pathway and suppress autophagy, which adds another layer of complexity for ErbB2 to regulate the mTOR pathway, leading to autophagy (Fig. 4D).

## Methods

### Cell culture and chemicals

SK-BR3, BT474, AU565, MCF7, and MDA-MB-361 cells were obtained from American Type Culture Collection (ATCC). All the cell lines were authenticated by the ATCC, and were expanded and preserved in liquid nitrogen upon receipt. Cells for experiments were passaged for fewer than 25–30 times. SK-BR3, MCF7, and MDA-MB-361 cells were maintained in Dulbecco’s modified Eagle medium (DMEM) supplemented with 10% (v/v) fetal bovine serum (FBS) and 1% (v/v) penicillin-streptomycin (PS) at 37 °C in a 5% CO_2_ humidified incubator. BT474 and AU565 cells were maintained in Roswell Park Memorial Institute (RPMI) 1640 medium supplemented with 10% FBS and 1% PS. Mubritinib (TAK-165, HY-13501) and HRGβ1 (HY-P7365) were purchased from MedChem Express.

### Western blotting

Cells were lysed in lysis buffer in the presence of protease inhibitors and phosphatase inhibitors. Cell lysates were then subjected to western blotting as previously described^41^. Primary antibodies were used as follows: p-ErbB2 (Y1221/1222) (2243#), ErbB2 (2165#), DEPTOR (11816#), p-AKT (S473) (4060#), p-AKT (T308) (4056#), t-AKT (4691#), p-S6K1 (T389) (9234#), p-ERK (T202/Y204) (9101#), t-ERK (4696#), mTOR (2972#), PARP (9532#), and LC3B (2775#) (Cell Signaling Technology); t-S6K (sc-230#), and ErbB2 (sc-33684#) (Santa Cruz); Tubulin (T9026#), LC3B (L7543#), and ACTIN (A5441#) (Sigma); p62 (PM045#) (Medical & Biological Laboratories).

### siRNA transfection

Cells were transfected with siRNA oligos in 60-mm dishes or 6-well plates using Lipofectamine 2000, according to the manufacturer’s instructions (Invitrogen). The sequences of siRNA oligonucleotides were as follows: siErbB2-1: 5′-GCAGTTACCAGTGCCAATA-3′; siErbB2-2: 5′-AAATTCCAGTGGCCATCAA-3′; simTOR-1: 5′-AAGAATCAAAGAGCAGAGTGC-3′; simTOR-2: 5′-GCTGTGCTACACTACAAACAT-3′; and siCtrl: 5′-ATTGTATGCGATCGCAGAC-3′.

### DNA transfection and dual-luciferase assay

The luciferase reporter driven by *DEPTOR* promoter was generated by PCR amplification using pfx DNA polymerase (Invitrogen) with the following primers: DEPTOR-luc-F: 5′-AGATCTGGTACCGAGGATAAAGTGTTTGGCACAATGT-3′ and DEPTOR-luc-R: 5′-AGATCTCTCGAGGCTGTAAGCCGAGTTCGGGT-3′. The PCR products were subcloned into the KpnI and XhoI sites of pGL3 luciferase reporter, subsequently verified by Sanger sequencing. Cells were transfected with 1 µg of luciferase reporter constructs, along with 0.2 µg of Renilla construct, using Lipofectamine 3000 following the manufacturer’s instructions (Invitrogen). Luciferase activity was measured using the Promega Dual-Luciferase Reporter Assay System kit (E1910, Promega), following the manufacturer’s instructions. The relative firefly luciferase activity was normalized to Renilla luciferase activity.

### Immunofluorescence staining

For immunofluorescence staining, the cells were first fixed with 4% formaldehyde for 15 min and then treated with 0.05% TritonX-100 for 10 min. Next, the cells were blocked for 30 min, and stained with anti-ErbB2 antibody (1:500) or anti-LC3B antibody (1:500, Sigma) for 1 h, followed by staining with secondary antibodies conjugated with Alexa Fluor 488 (1:500, Abcam) for 30 min and DAPI (1:1000, Beyotime) for 10 min at room temperature. The cells were then photographed under a confocal fluorescence microscope (Nikon).

### Subcellular fractionation

Cell fractions were extracted using Cell Fractionation Kit (9038#, Cell Signaling Technology), according to the manufacturer’s instructions. Briefly, cells harvested by trypsin were lysed in cytoplasm isolation buffer containing protease inhibitors and 1 mM PMSF, vortexed for 5 s at ultrahigh speed to fully resuspend, and then incubated on ice for 5 min, followed by centrifugation at 500 *g* for 5 min at 4 °C. The supernatants were transferred to a clean tube as cytoplasmic fractions. The pellets were lysed in membrane isolation buffer containing protease inhibitors and 1 mM PMSF, vortexed for 15 s at ultrahigh speed to fully resuspend, and then incubated on ice for 5 min, followed by centrifugation at 8000 *g* for 5 min at 4 °C. The supernatants were saved as membrane and organelle fractions. The pellets were ultrasound lysed in cytoskeleton/nucleus isolation buffer containing protease inhibitors and 1 mM PMSF, at 60% of power for 5 min at 4 °C, followed by centrifugation at 13,600 rpm for 5 min at 4 °C. The supernatants were collected as cytoskeletal and nuclear fractions.

### Chromatin immunoprecipitation (ChIP)

The ChIP assay was performed using the Simple ChIP Enzymatic Chromatin IP Kit (9003#, Cell Signaling Technology) according to the manufacturer’s instructions. The primer sequences for DEPTOR and COX-2 were as follows: DEPTOR-F: 5′-ATACTGCCATAAACATTACTTCGCC-3′ and DEPTOR-R: 5′-GGTATTGTCTAT CCGTAAAAGATTATGAA-3′; COX-2-F: 5′-CTTCAAAATAAGCTTGAATTCAGGATTGTAATG-3′ and COX-2-R: 5′-CTTTTTGATAATTTAATAATTTCAATCTTCTGTTTC-3′; DEPTOR-upstream-F: 5′-AGGAGACCTACAAGCATTTCGTG-3′ and DEPTOR-upstream-R: 5′-TTCATTTCCAACCCTGCTCAC-3′.

### Quantitative RT-PCR

Quantitative RT-PCR analysis was performed as described previously^42^. Briefly, total RNA was extracted from cells using TRIzol reagent (15596018, Invitrogen). cDNA was synthesized from RNA using the PrimeScript RT reagent kit (RR037A, Takara). Quantitative real-time PCR was accomplished using SYBR Premix Ex Taq (RR420A, TaKaRa) on an Applied Biosystems StepOnePlus^TM^ Real-Time PCR instrument. The primer sequences were as follows: DEPTOR-F: 5′-GCAGCAGGAATGAAGGTCTG-3′ and DEPTOR-R: 5′-GTATGTGCGGAGAAGACTCGTAT-3′; GAPDH-F: 5′-AGGGCATCCTGGGCTACAC-3′ and GAPDH-R: 5′-GCCAAATTCGTTGTCAT ACCAG-3′.

### Transmission electron microscopy

BT474 and MDA-MB-361 cells cultured in 60-mm dishs were collected by trypsin. Cells were rinsed with 0.1 M phosphate buffer (pH 7.4) before fixing with 2.5% glutaraldehyde in phosphate buffer at 4 °C overnight, and then post-fixed in 1% Osmic acid at room temperature for 1–2 h. After ethanol and acetone dehydration, penetrant treating and embedding in polybed 812 resin, thin sections (70 nm) were post-stained with 2% uranyl acetate followed by 0.3% lead citrate for 10 min. The photos of sample sections were taken using a TECNAI 10 transmission electron microscope (FEI Company, Hillsboro, OR) at 120 kV. To quantify autophagic vacuoles, five micrographs were taken with systematic random sampling from each sample.

### Correlation between the expression of ErbB2 and DEPTOR in breast cancer

Gene-level expression data (in format of FPKM) of ErbB2 and DEPTOR from the TCGA-BRCA project in The Cancer Genome Atlas (TCGA) were downloaded from the GDC data portal (https://portal.gdc.cancer.gov/). FPKM refers to Fragments Per Kilobase of transcript per Million mapped reads. A total of 83 ErbB2-positive BRCA tumor samples with FPKM of ErbB2 over 200 were included in the analysis. Pearson’s correlation between the expression of ErbB2 and DEPTOR was calculated in these 83 BRCA tumor samples.

### Statistical analysis

The data from three independent experiments were expressed as the mean ± SEM and analyzed using GraphPad Prism 5. The comparison of parameters between groups was performed using the two-tailed Student’s *t*-test with SPSS 20.0 (IBM). *p* < 0.05 was considered statistically significant.
